# Unstable mitochondrial heteroplasmy in *Mytilus edulis* primary cell cultures

**DOI:** 10.7717/peerj.21530

**Published:** 2026-07-02

**Authors:** Hajar Hosseini Khorami, Ricardo Fong-Zazueta, Sophie Breton, Annie Angers

**Affiliations:** Department of Biological Sciences, University of Montreal, Montreal, Quebec, Canada

**Keywords:** Heteroplasmy, qPCR, Cell culture, Germ cells, Doubly uniparental inheritance, Blue mussels, *Mytilus*

## Abstract

Doubly uniparental inheritance is a phenomenon unique to bivalve mollusks characterized by the inheritance of mitochondria from both parents by male embryos and the maintenance of paternal mitochondria in male gonads. This lineage-specific mechanism has been described in several bivalve species, where male mitochondrial DNA is also often found in somatic tissues. We exploited the development of a primary cell culture model for the blue mussel *Mytilus edulis* to investigate mitochondrial DNA dynamics at the cellular level and over time, using cells dissociated from male and female mantle (gonadal) and gill (somatic) tissues. We employed quantitative polymerase chain reactions to monitor fluctuations in the relative abundance of male and female mitochondrial DNAs. In male mantle cells, the amount of male mtDNA increased over time in culture across all tested samples. In male gill cells, male mtDNA was detectable in approximately 50% of the samples at the initial time point and generally decreased over time. No heteroplasmic female could be detected in this study. Illumina sequencing, comparing the proportion of the sex-linked mtDNAs between male mantle tissue and derived cultured cells, revealed a sharp increase in the proportion of male mtDNA in cultured mantle cells. Finally, immunofluorescence experiments using antibodies against open reading frames (ORFs) encoded by male or female mtDNA genomes showed that these peptides are present in the mitochondria of all male mantle cells after 10 days in culture. Overall, these findings support the idea that heteroplasmy is maintained in male germ cells in culture, whereas heteroplasmic cells in somatic tissues tend to be scarce and are lost over time.

## Introduction

Mitochondria are organelles of endosymbiotic origin and, as such, have maintained a distinct genome. In most bilaterian animals, mitochondrial DNA (mtDNA) is usually a circular molecule of about 16 kb that encodes 22 tRNAs, two rRNAs, and 13 polypeptide subunits of the oxidative phosphorylation complexes I, III, IV, and V (Wolstenholme & Clary, 1985; Boore, 1999). Many copies of mtDNA are present per cell in somatic and germ cells, and mutations can be present in a fraction of any mtDNA population. The presence of a mixture of mtDNA is termed heteroplasmy (Larsson & Clayton, 1995). In most animals, mtDNA is exclusively maternally inherited due to the downregulation of mtDNA copy number during spermatogenesis and active degradation of sperm mitochondria after fertilization, effectively reducing heteroplasmy at the population level (Sato & Sato, 2017; Lee et al., 2023). Strict maternal inheritance of mtDNA is thought to have evolved to help maintain homoplasmy across generations, as the rare occurrence of paternal mitochondria in humans is associated with severe symptoms (Schwartz & Vissing, 2002; Luo et al., 2018). In *Caenorhabditis elegans*, the loss of *cps-*6 is associated with a slight delay in paternal mitochondria elimination at the embryonic stages, which leads to important physiological impairments in adult animals (Zhang, Zhu & Xue, 2024). Nevertheless, several bivalve mollusk species have been shown to escape this general rule and actively maintain a distinct paternally-transmitted mtDNA molecule in their germinal cell lineage (Zouros et al., 1994; Breton et al., 2014; Gusman et al., 2016). In these species, the maternal (F) mtDNA is passed to both male and female individuals. At the same time, the paternal (M) mtDNA is lost/eliminated in females but retained in male individuals’ gametes. Since the F-mtDNA is inherited only from the maternal lineage and the M-mtDNA is passed from fathers to sons, this mode of transmission has been named “doubly uniparental inheritance” (DUI) (Zouros et al., 1994). This independent transmission of the two genomes has resulted in the co-habitation of two highly divergent mtDNA molecules within the same species (Zouros, 2013).

The origin and consequences of DUI are still unclear, although many hypotheses have been advanced (reviewed in Parakatselaki & Ladoukakis, 2021; Breton et al., 2022). The discovery of sex-specific mtDNA-encoded proteins (female-specific open reading frame (F-ORF) and male-specific open reading frame (M-ORF)), and the correlation of their presence with gonochorism and DUI has led to the hypothesis that the specific F and M-mtDNA genomes might be linked to sex determination in species with DUI (Breton et al., 2009; Breton et al., 2011). In the blue mussel *Mytilus edulis*, F-ORF is observed in most cell types, while M-ORF is strictly present in male germ cells’ mitochondria and mature sperm mitochondria and acrosome (Brémaud et al., 2025).

Quantitative polymerase chain reaction (qPCR) analysis in *Mytilus* spp. and the marine clam *Ruditapes philippinarum*, also a species exhibiting DUI, suggested that female somatic tissues are mostly homoplasmic for the F-type, whereas male somatic tissues are mostly heteroplasmic (Ghiselli, Milani & Passamonti, 2011; Obata, Sano & Komaru, 2011; Milani et al., 2014a; Milani et al., 2014b). The extent of such somatic heteroplasmy in males depends on tissue and species (Ghiselli, Milani & Passamonti, 2011; Zouros, 2013; Milani et al., 2014a; Milani et al., 2014b). In contrast, strict homoplasmy seems to be reached in mature gametes of both sexes through mechanisms that are still to be elucidated (Ghiselli et al., 2019).

Despite the wide range of studies on heteroplasmy in DUI species, previous research has been mostly based on dissected tissues, which preclude continuous, long-term experiments on the same sample. In contrast, cell culture-based studies enable repeated measurements over time, facilitating prolonged investigations on a consistent biological model and allowing real-time monitoring of mitochondrial dynamics under controlled conditions. This approach focuses more precisely on selected cell types while reducing the complexity associated with tissue-based studies. In a previous study, the success of primary cell cultures derived from gonadal (mantle) and somatic (gill) tissues of M. edulis was reported (Hosseini Khorami, Breton & Angers, 2024). The designed primary culture conditions supported the long-term survival, proliferation, and *in vitro* differentiation of dissociated primordial germ cells from males, making these cultures well-suited for investigating heteroplasmy at the cellular level. Here, we utilize this culture system to assess heteroplasmy levels in cells derived from various tissues, examining the maintenance of M- and F-type mtDNAs over time in culture. As we hypothesized that male germ cells proliferate better than somatic cells in this system, and since they are the precursors of homoplasmic gametes, we expected that M- and F-type mtDNA would be differentially maintained in cultures from male mantle.

## Materials & Methods

### Cell cultures

Freshly harvested *M. edulis* from Prince Edward Aqua Farm, Canada, were purchased from the market and brought to the lab on ice within 24 h before starting the cultures. In brief, to determine the sex of each blue mussel, the dissected mantle tissue was placed in a 35 × 10 mm non-treated Petri dish containing artificial seawater (ASW) for 5 min. A 1 µL sample of the Petri dish’s contents was examined under an optical microscope to identify mature eggs or active sperm. Only individuals with clearly identified mature gametes were chosen for the experiments. Mantle was selected as a cell source since it contains the gametes in *M. edulis* and gill was chosen as a source of somatic cells for comparison. Heteroplasmy is sometimes observed in the gill of *Mytilus* (Obata, Sano & Komaru, 2011)*.* The cell dissociation protocol was carried out on the selected tissues following the method suggested by Hosseini Khorami, Breton & Angers (2024). To prevent contamination of somatic tissues by sperm in male samples and to avoid cross-tissue contamination, (1) separate dissection instruments were used for each animal and tissue type, (2) gills were dissected at distance from the inner shell edge and mantle tissues, and (3) each tissue was kept in an individual plate containing artificial seawater (ASW) plus antibiotics and washed extensively by changing plates and ASW supplemented with antibiotics. Then, cells of each individual/tissue were cultured seeded at approximately 2  ×  10^4^ cells/well in 24-well plates pretreated with poly-D-lysine and fed with a complete media composed of artificial seawater enriched with L-15 and 20% bovine calf serum. A combination of antibiotics (penicillin (100 U/mL), streptomycin (100 µg/mL), and amphotericin B (1.5 µg/mL)) was added to the feeding media. The incubator temperature was set to 23 ± 2 °C, and the media was refreshed every 5 days (Hosseini Khorami, Breton & Angers, 2024).

Dissociated cells of the mantle and gill tissues of six mature males and three mature females were seeded in 24-well plates (nine wells per individual for each tissue). For qPCR analysis, cells from three wells were collected for DNA extraction after 1, 5 or 10 days in culture (Day 1, Day 5, and Day 10). Experiments were run during *Mytilus*’s two main spawning seasons, December (five males and all females) and April (one male). For the sequencing experiment, part of the mantle tissue from a mature male was dissociated, and cells were cultured for one day. DNA was extracted from the cultured cells and the remaining tissue (Day 0).

### Sample collection and DNA extraction

At the indicated time cells were harvested using cell scrapers, transferred to Eppendorf tubes, and centrifuged at 6000 ×g. The supernatant was discarded, and pellets were kept at −80 °C. Total DNA was extracted from cultured cell samples using the QIAamp UCP DNA Micro Kit (Qiagen 56204) following the manufacturer’s protocol. The concentration, quality, and purity of the extracted DNA were evaluated using a BioDrop µLITE spectrophotometer.

### Quantification of heteroplasmy by quantitative polymerase chain reaction

Quantitative polymerase chain reaction (PCR) assays targeted the male-type cytochrome b (M-cytb), female-type cytochrome b (F-cytb), and 28S rRNA (nuclear) genes. M- or F-mtDNA was quantified relative to 28S rRNA. Primers used for these assays were previously validated by Obata, Sano & Komaru (2011) and Sano, Obata & Komaru (2010) and are presented in Table 1 and Fig. S1. To assess primers’ suitability and optimize their concentration for this study, a two-fold serial dilution of DNA in the reaction (ranging from 10 to 0.156 ng) was performed to design a standard curve and ensure target amplification efficiency.

All qPCR assays were run in triplicate in a 20 µl total volume, using a Thermo Scientific PikoReal 96 Real-Time PCR instrument and Power SYBR Green PCR Master Mix (Thermo Fisher 4367659) as a universal formulation. Each qPCR reaction contained Power SYBR Green PCR Master Mix (10 µL), forward and reverse primers (0.3 µM final concentration for the 28S rRNA primer set or 0.4 µM final concentration for the M- and F-cytb primer sets), and ultrapure distilled water (Thermo Fisher) to adjust the final reaction volume to 20 µL, as needed. 10 ng/µL of template DNA was added as the final component. The thermal protocol consisted of 2 min at 50 °C and 10 min at 95 °C for an initial denaturation and activation of Taq polymerase, followed by 40 cycles of 15 s at 95 °C and 1 min at 60 °C). The melting curve was checked to examine the quality of the amplification and lack of mispriming. A single sample presenting multiple melting curves was discarded. Another sample had to be discarded because of obvious manipulation errors (inverted melting curves and unlabeled wells) (Fig. S2).

The initial level of M-mtDNA in each experimental model was normalized to the reference gene using the -ΔC formula (Schmittgen & Livak, 2008). To examine the temporal regulation of sex-linked mitochondrial abundance in mantle and gill tissues *in vitro*, the mtDNA level was normalized to the reference gene at related timepoints and then folded to Day 1 following 2^−ΔΔCt^ formula (Schmittgen & Livak, 2008). No-template controls (NTCs) were included for each primer pair in every qPCR run. Sample CT values were considered quantifiable when they fell at least three cycles below the corresponding non-template-containing (NTC) reactions CT (Table S1).

**Table 1:** Primers used in qPCR experiments.

Target gene	Primers
Male type cytochrome b (M-cytb) (Obata, Sano & Komaru, 2011)	Forward 5′-AGGTTTGGGTCTATACTAGGTTTAAGGT-3′
	Reverse 5′-TCGTGGGCAGTATAGTGAATTGAC-3′
Female type cytochrome b (F-cytb) (Obata, Sano & Komaru, 2011)	Forward 5′-TAATTCCTACGCTTCATACAGGTAAGTAC-3′
	Reverse 5′-TTCATGTTAGGCTAATAAACCTTCCA-3′
28S ribosomal RNA (28S rRNA) (Sano, Obata & Komaru, 2010)	Forward 5′-CAAGAGTACGTGAAACCGCTTAGA-3′
	Reverse 5′-CCGACAACGACAAGTTGAATTC-3′

### Illumina NovaSeq sequencing

Illumina Nova Seq sequencing was used to compare the reads mapping to the M-mtDNA or F-mtDNA in male mantle compared to the cells in culture. DNA was obtained as previously described and resuspended in 10 mM Tris–HCl (pH 8.0) with 0.1 mM EDTA, as recommended by Génome Québec, to prevent degradation. Samples were then sent to Génome Québec for sequencing using Illumina NovaSeq technology. The trimming and mapping parameters consisted of quality control with fastqc (Andrews, 2010), trimming with fastp v-0.24.0 (Chen et al., 2018), mapping with Burrows Wheeler Aligner (BWA) v-2.2.1 (Li & Durbin, 2009) with the mem algorithm, and filtering for high-quality mapping reads with Samtools v-2.2.1 (Li et al., 2009; Danecek et al., 2021). The mapping was performed against both *M. edulis* haplotype 1 mitochondrion complete genome (AY823623) for male mitochondrial DNA (Breton et al., 2006) and *M. edulis* mitochondrion complete genome (AY484747) for female mitochondrial DNA (Hoffmann, Boore & Brown, 1992) at the same time, allowing the fragments to map against the genome to which their sequence is more similar.

### Immunohistochemistry

Immunocytochemistry analyses were conducted as in Hosseini Khorami, Breton & Angers (2024) on cells cultured from mantle tissues of four male specimens after 10 days in culture. Briefly, cells were seeded on poly-D-lysine-coated coverslips. After washing with 1X phosphate buffered saline (PBS PH 7.4 ± 0.1, 1.8 mM KH_2_PO_4,_ 10 mM Na_2_HPO_4,_ 137 mM NaCl, and 2.7 mM KCl), cells attached to each coverslip were fixed with 4% paraformaldehyde for 30 min, rinsed 3 times with PBS, and permeabilized with 0.2% triton X-100 for 4 min. Blocking was conducted for 30 min with 5% bovine serum albumin (BSA) mixed with 5% normal goat serum (NGS), washed three times with PBS, and incubated overnight in a humid chamber with 1:2000 anti-ATP5A antibodies (Abcam, ab14748) to label mitochondria and either anti- M-ORF (Debelli et al., 2023) or F-ORF (Ouimet et al., 2020) both at 1:200. Both antibodies were further validated in Brémaud et al. (2025). After washing again with PBS, secondary antibodies were applied for one hour at room temperature. Goat anti-Mouse IgG (H+L) Alexa Fluor™ 594 and Goat anti-Rabbit IgG (H+L) Alexa Fluor™ 488 (Invitrogen) were used. Finally, after another series of washes in PBS, the slides were mounted with Fluoroshield Mounting Medium with DAPI (Abcam ab104139), and imaged (EVOS M5000). Omitting the secondary antibodies was used to ascertain the specificity of the staining (not shown). Five randomly chosen frames were captured for each coverslip to count the number of cells positive for M-ORF or F-ORF signals.

### Statistical model

Mcytb expression was analyzed using the multivariable linear model (MLM) approach proposed by Hampton et al. (2025), which has the advantage of not assuming an amplification efficiency of two or identical efficiencies for the target and reference genes. Raw CT values were modelled directly as: *CT*_*target*_ ∼ *CT*_*ref*_ + time + individual where *CT*_*ref*_ (28SrRNA) is treated as a continuous covariate, time is a three-level factor (Day 1, Day 5, Day 10), and individual is included as a factor to account for the paired data structure (*n* = 5 repeated measures). The estimated slope associated with *CT*_*ref*_ (*β* ≈  − 0.03) indicates similar amplification efficiencies between the two genes, and the results are therefore consistent with those obtained by the classical 2.11Ct/ method.

## Results

### Relative levels of sex-linked mtDNAs in mussels’ primary cell cultures

**Figure 1:**
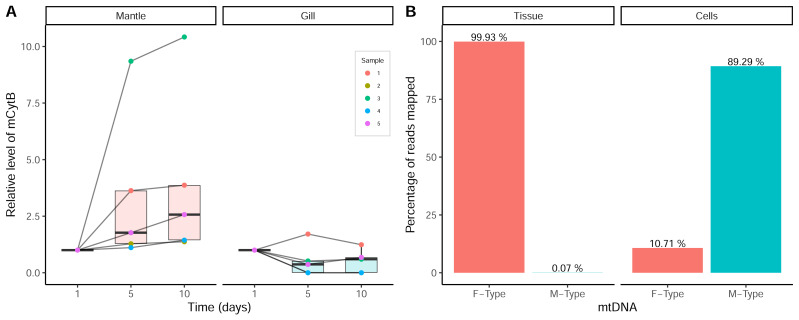
Relative change in abundance of M-type mtDNA in cultured cells. Comparison of average mitochondrial DNA (M-mtDNA) abundance levels in male mantle and gill cell cultures over time. M-mtDNA levels, specifically M-cytb, relative to the reference gene (28S rRNA), were quantified using quantitative PCR (qPCR) from total DNA extracted from cells cultured from male mantle and gill samples at days 1, 5, and 10. In all experiments, dissociated cells were cultured in complete media and harvested at the indicated time. qPCR analyses were performed in triplicate for each sample. Statistical analysis showed no significant differences in average M-mtDNA abundance levels across the timepoints within each tissue group. Colors identify individuals and correspond to the raw data presented in Table S1. (B) Illumina reads mapping to the sex-linked mitochondrial genomes in DNA extracted from mantle tissue or dissociated cells in culture. The bars show the proportion of reads uniquely mapping to F-mtDNA (ref. AY484747.1 or M-mtDNA (ref. AY823623.1).

To assess the modulation of heteroplasmy over time in cultured cells, we performed quantitative PCR (qPCR) analysis of M-mtDNA levels in primary cultures of mantle and gill tissue from six males and three females. (all data available in Supplementary File 1). M-mtDNA levels were above background in only one female sample, but this sample had to be discarded due to the presence of multiple melting peaks (Table S1). Female cells were thus not considered further. All male mantle samples were positive for M-cytb (Table S1). On average, the relative level of M-mtDNA increased approximately 3-fold in male mantle cells from day 1 to day 10. However, the high level of heterogeneity only allowed for statistical significance in relative M-mtDNA abundance when Day5 and Day10 together were compared to Day1 (Wilcoxon signed-rank test, *p* = 0.0312, mean FC = 2.66 ×, *d* = 1.14). Since the sample size limits the statistical power, we examined each sample individually to understand the evolution of heteroplasmy over time across individuals (Fig. 1A). qPCR results for primary cell cultures derived from male mantle samples exhibited a consistent pattern across all samples, with the relative abundance of M-mtDNA increasing from day 1 to day 10. In three out of five males, the increase was particularly pronounced, ranging from 2.5 to 10 times. A more subtle increase was observed in the remaining two samples, approximately 1.5 times. The highest rise in the relative M-mtDNA abundance was seen in male 3, in which an increase of ∼10.5-fold was recorded.

qPCR analysis of primary cell cultures derived from male gill samples revealed that four out of five samples were positive for the presence of M-mtDNA on day 1. When mtDNA was present at the beginning of the experiment, it either remained stable or tended to disappear (Fig. 1A, Table 2 and Table S1). Thus, unlike male germ cells, gill cells do not retain M-mtDNA in culture.

**Table 2:** Temporal changes in relative M- and F-mtDNA abundance in male mantle and gill cell cultures (2^−ΔΔCt^ normalized to day 1).

		Fold changes in M-mtDNA			Fold changes in F-mtDNA		
Individual	Tissue	Day 1	Day 5	Day 10	Day 1	Day 5	Day 10
Male 1	Mantle	1	3.63	3.87	1	2.13	6.25
	Gill	1	1.71	1.24	_	_	_
Male 2	Mantle	1	1.28	1.37	1	0.81	1.50
	Gill	1	1.11	2.33	_	_	_
Male 3	Mantle	1	9.35	10.42	1	1.04	5.33
	Gill	1	0.52	0.60	_	_	_
Male 4	Mantle	1	1.11	1.44	_	_	_
	Gill	1	0.10	0	_	_	_
Male 5	Mantle	1	1.77	2.57	_	_	_
	Gill	1	0.37	0.68	_	_	_

Because differentiated gametes have been shown to be homoplasmic for M and F mitochondria, we wondered if the level of F-mtDNA would also change with time in male mantle cell cultures. We previously demonstrated that the abundance of germ cells increased over time in culture; however, the cells did not fully mature into spermatozoa (Hosseini Khorami, Breton & Angers, 2024). The relative abundance of F-mtDNA in male mantle samples demonstrated the presence of F-mtDNA alongside M-mtDNA at all timepoints. The amount of F-mtDNA also increased over time, suggesting that male germ cells remain heteroplasmic, at least at the level of differentiation that occurs after 10 days in culture (Table 2 and Table S1).

Illumina sequencing revealed distinct distributions of M-mtDNA and F-mtDNA in male mantle tissue and cultured cells. In the tissue sample, less than 1% of total mitochondrial DNA reads mapped to M-mtDNA, and almost ∼90% allocated to F-mtDNA. Conversely, cultured cells showed a reverse pattern with reads mapped to F- and M-mtDNA being ≈10% and ≈80%, respectively. Typically, reads corresponding to sequences that are identical in both M- and F-mtDNA remained constant (9.62% in tissue samples and 10.74% in cultured cells) (Fig. 1B).

### Expression of M- and F-mtDNA encoded proteins in male mantle primary cell cultures

**Figure 2:**
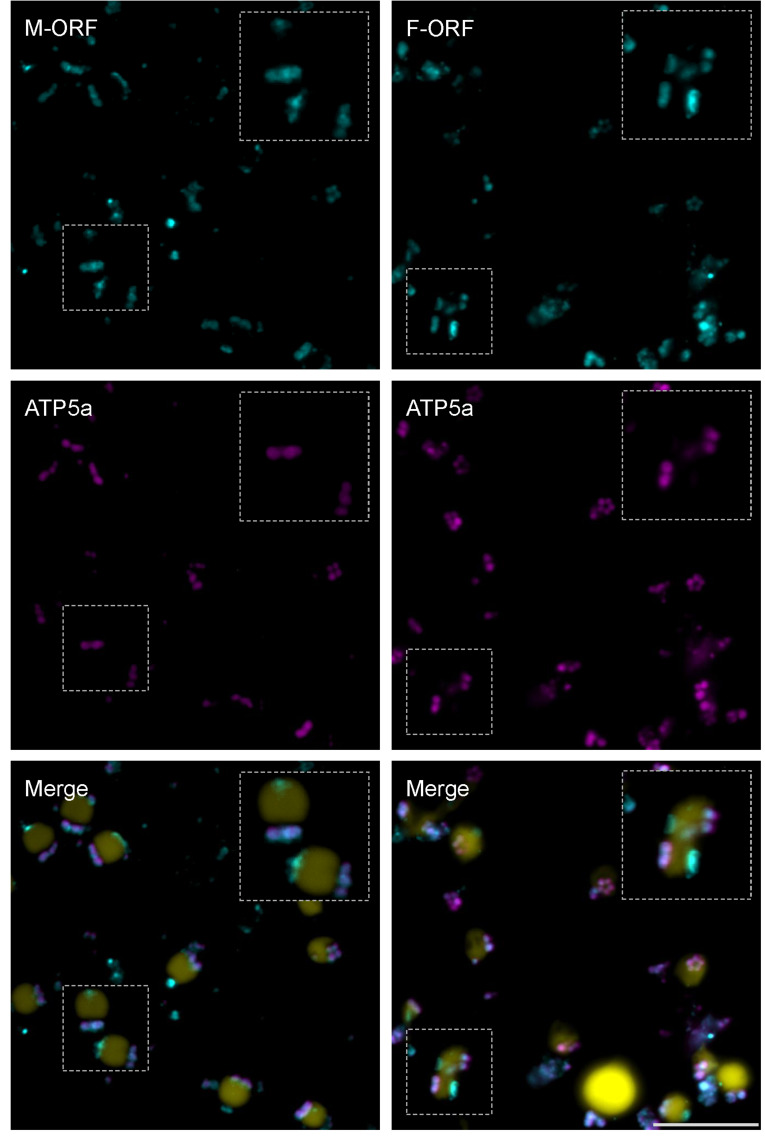
Immunofluorescence labelling of M-ORF, F-ORF, and ATP5A in cultures originating from male mantle cells. Cells dissociated from the mantle tissue (previously identified as germ-cell-rich) of four male specimens were cultured in complete media. Immunofluorescence staining for M- and F-ORF (cyan) and ATP5A (magenta) was performed, with nuclei counterstained with DAPI (yellow) on day 10. The figure shows six representative images arranged in three rows and two columns. Each column represents a separate culture well plate from the same tissue: the right column shows M-ORF-stained cells, and the left column shows F-ORF-stained cells. In each column, the top row shows the ORF channel, the middle row shows ATP5A, and the bottom row shows the merged image of ORF, ATP5A, and DAPI. Within each panel, a subset of cells is highlighted with a dotted box in the lower-left corner, and the same cells are shown enlarged in a second dotted box in the upper-right corner to better visualize subcellular localization. Images were captured using the EVOS-M5000 fluorescent microscope. Images are representative examples of five captured frames at 100× magnification in four independent experiments on day 10. The scale bar represents 10 µm.

The M- and F-mtDNA genomes of *M. edulis* each contain gender-specific open reading frames (ORFs) of uncertain origin that are expressed in mitochondria (Ouimet et al., 2020; Debelli et al., 2023). We performed immunohistochemistry experiments to determine if the number of cells expressing M-ORF and F-ORF in male mantle cell culture changed over time, as the amount of M-mtDNA increased. The analysis revealed that all male mantle cells imaged expressed M-ORF at every time point. F-ORF, a protein encoded by the F-mtDNA, was also consistently expressed in these cells, consistent with a heteroplasmic state (Fig. 2). In all cells, M-ORF and F-ORF signals overlapped with ATP5A, a mitochondrial marker. In addition, both peptides were also detected in the forming acrosomes (Fig. 2).

## Discussion

In the context of the DUI system, male individuals are typically described as heteroplasmic, with the M-mtDNA found exclusively in their gametes and the F-mtDNA present throughout their somatic tissues. In contrast, female individuals are generally considered homoplasmic for the F-mtDNA. However, heteroplasmy has been observed in both male and female somatic tissues of various species, including marine mussels *Mytilus* spp. (order Mytiloida), the marine clam *Ruditapes philippinarum* (Veneroida) and freshwater mussels (Unionoida) (Zouros, 2013; Milani et al., 2014a; Milani et al., 2014b; Breton et al., 2017). In the present study, we aimed to further investigate the dynamics of heteroplasmy in a cell culture system derived from *M. edulis*, rather than in whole tissues, which have been the predominant focus of previous research. Using qPCR and ICC techniques, we tracked the stability of M-mtDNA in cells obtained from somatic tissues (gills) and germline tissues (mantle during spawning season) of mature *M. edulis* individuals over time.

Globally, our results indicate that male mantle cells, that we established to be mainly germ cells (Hosseini Khorami, Breton & Angers, 2024), tend to maintain their M-mtDNA *in vitro*, exhibiting either stable or notably positive fluctuations. In contrast, most gill samples were unable to preserve their heteroplasmic state over time in culture. Conversely, we did not find a heteroplasmic female in this study.

A likely explanation for the two different patterns of heteroplasmy observed *in vitro* is that male mitochondria confer a proliferation advantage to male germ cells but not to other cell types. This observation could be linked to the “father’s curse” hypothesis, which posits that M-mtDNA is beneficial in sperm but is regulated by a maintenance system that applies different strategies to mitigate the detrimental effects of heteroplasmy in somatic tissues or eggs. For example, although M-mtDNA is sometimes transcribed in the somatic tissues of the freshwater mussels *Venustaconcha ellipsiformis* and *Utterbackia peninsularis* , as well as the marine clam *R. philippinarum* , it is suggested that it might be silenced (*i.e.,* not translated) or downregulated to prevent the negative consequences of heteroplasmy (Milani et al., 2014a; Milani et al., 2014b; Breton et al., 2017). Notably, we previously demonstrated that the M-specific ORF of *M. edulis* mtDNA is only expressed during the mating period in male mantles, indicating that it may not be translated or present in very low abundance outside germ cells (Debelli et al., 2023).

Another hypothesis for managing heteroplasmy involves assigning different roles and metabolic functions to the two sex-linked mitotypes (Breton et al., 2017). This idea was explored in *R. philippinarum* (Milani et al., 2014a; Milani et al., 2014b) and *M. edulis* (Bettinazzi et al., 2019). For instance, mitochondrial respiratory analyses of gametes and somatic tissues of male and female *Mytilus* revealed a metabolic remodelling in sperm mitochondria. These changes are thought to represent a strategy for minimizing conflicts arising from the coexistence of two divergent mitotypes in the same nuclear background, thereby ensuring cellular survival and optimal cell performance. Since this “M mitochondrial phenotype” was also partly observed in male gills heteroplasmic for both M- and F-type mtDNAs, it was suggested that somatic tissues can tolerate heteroplasmy as long as the level of M-mtDNA does not exceed a critical threshold that causes a harmful phenotype (Bettinazzi et al., 2019). In our study, somatic cells from male gills progressively lost their heteroplasmic states, even when the initial amount of M-mtDNA was relatively high. Speculatively, one could assume that although tolerated, M-mtDNA is not replicated as efficiently as F-mtDNA in cell-types other than male germ cells.

Building upon the hypothesis regarding the management of heteroplasmy, the maintenance of heteroplasmy in mantle cell cultures also leads us to hypothesize that the observation made by Ghiselli et al. (2019) of heteroplasmy at the organelle level (within mitochondria themselves) might extend to primordial germ cells. This further supports the idea that M-mtDNA and F-mtDNA are expressed in undifferentiated and premature male germ cells. Since F-mtDNA was well maintained in our experiments, we must conclude that the decrease and eventual loss of F- mtDNA only occurs at a maturation stage that is not reached in culture, at least not in 10 days. Either the necessary factor(s) is extrinsic and therefore eliminated by dissociation of the tissue, or it is intrinsic to germ cells but not expressed in dissociated cells. In any case, the heteroplasmic pattern remains clearly divergent between somatic cells and germ cells, establishing that cells cultured from male mantle tissue are of the germ-cell lineage.

To further explore the mitochondrial DNA composition in both mantle tissue and cultured mantle cells, we performed Illumina sequencing. The results were consistent with our qPCR findings, revealing a shift in mtDNA composition, with cultured mantle cells exhibiting a dramatically higher proportion of M-mtDNA reads. Mantle tissue contains both somatic and germ cells during the reproductive season, which explains the high proportion of F-mtDNA reads in the tissue sample. This disparity between the tissue and the cultured cells that derive from it is consistent with our previous work, which showed that male germ cells are better adapted to the culture system (Hosseini Khorami, Breton & Angers, 2024), and is further supported by the current qPCR results indicating the presence of M-mtDNA in these cells. Additionally, the hypothesis that male germ cells predominantly maintain M-mtDNA during maturation (Milani et al., 2014a; Milani et al., 2014b; Breton et al., 2014; Ghiselli et al., 2019) further supports our interpretation of the Illumina sequencing results.

Finally, the immunocytochemistry analysing the expression of M-ORF and F-ORF also supports the coexistence of M- and F-mtDNAs in male germ cells since all male mantle cells examined in culture consistently expressed both M-ORF (encoded by M-mtDNA) and F-ORF (encoded by F-mtDNA) proteins. As F-ORF is still present in mature sperm, further investigation is needed to determine how F-mtDNA decreases during the final stages of sperm maturation (Milani et al., 2014a; Milani et al., 2014b; Breton et al., 2014; Ghiselli et al., 2019; Debelli et al., 2023; Brémaud et al., 2025).

## Conclusions

In this study, we examined the presence and levels of male mitochondrial DNA (M-mtDNA) in established cell cultures derived from mantle (gonad) and gill (somatic) tissues of male *M. edulis* over a defined period. We found that, although the mantle is a heterogeneous tissue, germ cell precursors are selected during the culture process. These cells are highly heteroplasmic and remain so in culture. Conversely, when heteroplasmy is present in somatic tissues, it tends to decrease over time in culture. This suggests intrinsic differences between germ and somatic cells. How spermatocytes transition from heteroplasmic germ cells to homoplasmic gametes remains unresolved at this point, yet the culture system used here may provide an opportunity to directly test some hypotheses. As previously reported, male germ cells cultivated in bovine serum could not mature to spermatozoa (Hosseini Khorami, Breton & Angers, 2024). It would be interesting to measure heteroplasmy to determine whether a modified media composition could support spermatogenesis. Also, if, as suggested by the observation of proteins coded by male and female mtDNA in the same mitochondria of *Ruditapes philipinarum* (Ghiselli et al., 2019), both genomes coexist at the mitochondrial level, then it is necessary to selectively degrade F-type mtDNA to achieve homoplasmy in mature male gametes. This idea is consistent with observations of different methylation patterns in mtDNA of male and female gametes, and between spermatids and mature spermatozoa (Leroux et al., 2024). The *in vitro* system presented here provides a suitable model to further analyse the molecular details leading to sperm maturation and mtDNA dynamics in DUI species.

##  Supplemental Information

10.7717/peerj.21530/supp-1 Supplemental Information 1 Ct values for M-cytb and 28S in male and female primary cell cultures Ct (cycle threshold) values for M-cytb, F-cytb and 28S rRNA from experiments carried out on six male and three female *M.edulis* samples. Cells that have been considered zero are grayed out, except for the negative control column. The one occurrence of lower Ct values for M-cytb in Female 3 samples was associated with multiple melting peaks, indicating nonspecific amplification, and was therefore interpreted as the absence of M-mtDNA. All data points are the average of three replicates.

10.7717/peerj.21530/supp-2 Supplemental Information 2 MIQE Checklist

10.7717/peerj.21530/supp-3 Supplemental Information 3 Comarison of male and female mCytb gene in the region amplified in the qPCR experiements Partial sequence alignment of mCytB from F- and M-mtDNA, highlighting sequence divergence (grey shading) and the position of the primers used to amplify M-cytB and F-cytB in qPCR experiments.

10.7717/peerj.21530/supp-4 Supplemental Information 4 Melting curves (-dF/dT) for Mcytb and 28SrRNA across all qPCR samples. Raw derivative curves computed from the Melt Curve Data output of the qPCR instrument. Temperature range: 65-87 C. Blue lines: Mcytb target gene (wells A7-A12); red dashed: no-template control (NTC) for Mcytb (A13); green lines: 28SrRNA reference gene (D7-D12); grey dashed: NTC for 28SrRNA (D13). Panels outlined in orange indicate anomalous samples. M6: melting curve analysis revealed that the two primer pairs were loaded into transposed wells relative to all other individuals; wells could not be reassigned objectively due to an unexpected number of loaded wells. The sample was thus not considered in the analysis. F1: Mcytb wells showed broad non-specific amplification co-occurring with the NTC, leading to exclusion of this individual.

10.7717/peerj.21530/supp-5 Supplemental Information 5 Raw qPCR data Cq and melting curve values for all samples. Each sheet refers to a single individual.

## Additional Information and Declarations

### Data Availability

The following information was supplied regarding data availability: All the code and data used for the sequence analysis are available at Github and Zenodo: - https://github.com/RicardoFong/Mytilus_project
- Ricardo Fong Zazueta. (2026). RicardoFong/Mytilus_project: Unstable mitochondrial heteroplasmy in Mytilus edulis primary cell cultures (workflow-v1). Zenodo. https://doi.org/10.5281/zenodo.18840180
The qPCR raw data are available at Zenodo: - Angers, A., Hosseini Khorami, H., Breton, S., & Ricardo, F.-Z. (2026). qPCR experimental raw data set [Data set]. Zenodo. https://doi.org/10.5281/zenodo.20383018.
