# Supplementary material for: Unstable mitochondrial heteroplasmy in Mytilus edulis primary cell cultures
Source: PeerJ. 2026 Jul 2;14:e21530. doi: 10.7717/peerj.21530 (PMC13333129; doi:10.7717/peerj.21530)
Supplement: Supplemental Information 1 — Ct (cycle threshold) values for M-cytb, F-cytb and 28S rRNA from experiments carried out on six male and three female M.edulis samples. Cells that have been considered zero are grayed out, except for the negative control column. The one occurrence of lower Ct values for M-cytb in Female 3 samples was associated with multiple melting peaks, indicating nonspecific amplification, and was therefore interpreted as the absence of M-mtDNA. All data points are the average of three replicates. [file peerj-14-21530-s001.docx]

**Supplementary Table S1**

**Ct values for M-cytb and 28S in male and female primary cell cultures.** Ct (cycle threshold) values for M-cytb, F-cytb and 28S rRNA from experiments carried out on six male and three female *M.edulis* samples. Cells that have been considered zero are grayed out, except for the negative control column. The one occurrence of lower Ct values for M-cytb in Female 3 samples was associated with multiple melting peaks, indicating nonspecific amplification, and was therefore interpreted as the absence of M-mtDNA. All data points are the average of three replicates.

|  | Gene | Mantle | | | Gill | | | Negative  control |
| --- | --- | --- | --- | --- | --- | --- | --- | --- |
|  |  | Day 1 | Day 5 | Day 10 | Day 1 | Day 5 | Day 10 |  |
| Male 1 | M-cytb | 26.63 | 26.26 | 26.41 | 21.06 | 20.78 | 20.31 | 33.88 |
|  | F-cytb | 21.54 | 21.94 | 20.63 | - | - | - | 37.33 |
|  | 28S | 15.34 | 16.83 | 17.07 | 14.55 | 15.04 | 14.11 | 32.71 |
| Male 2 | M-cytb | 22.97 | 22.25 | 22.04 | 30.86 | 31.99 | 31.70 | 34.18 |
|  | F-cytb | 21.00 | 20.94 | 19.94 | - | - | - | 34.16 |
|  | 28S | 15.02 | 14.66 | 14.55 | 16.82 | 18.10 | 18.88 | 31.87 |
| Male 3 | M-cytb | 23.17 | 19.14 | 19.17 | 21.73 | 23.36 | 22.58 | 29.82 |
|  | F-cytb | 25.11 | 24.24 | 22.06 | - | - | - | NA |
|  | 28S | 14.08 | 13.27 | 13.45 | 16.08 | 16.77 | 16.20 | 32.80 |
| Male 4 | M-cytb | 21.69 | 20.35 | 19.78 | 30.68 | 31.64 | 33.88 | NA |
|  | 28S | 14.86 | 13.66 | 13.48 | 18.17 | 15.76 | 18.38 | NA |
| Male 5 | M-cytb | 20.91 | 19.99 | 19.16 | 25.86 | 27.51 | 26.49 | 35.21 |
|  | 28S | 14.47 | 14.37 | 14.08 | 16.37 | 16.60 | 16.44 | 32.25 |
| Female 1 | M-cytb | 35.55 | 34.74 | 35.87 | 35.54 | 34.98 | 34.21 | 34.06 |
|  | 28S | 17.77 | 14.13 | 13.24 | 16.79 | 14.61 | 13.74 | 29.75 |
| Female 2 | M-cytb | 38.09 | 38.12 | 38.17 | 37.75 | 38.81 | 37.67 | 37.70 |
|  | 28S | 14.89 | 17.46 | 13.83 | 15.06 | 14.75 | 16.99 | 29.91 |
| Female 3 | M-cytb | 29.53 | 27.43 | 27.67 | 28.11 | 28.16 | 30.68 | 30.59 |
|  | 28S | 17.43 | 14.56 | 13.43 | 16.97 | 15.36 | 19.57 | 31.13 |
