# Supplementary material for: Unstable mitochondrial heteroplasmy in Mytilus edulis primary cell cultures
Source: PeerJ. 2026 Jul 2;14:e21530. doi: 10.7717/peerj.21530 (PMC13333129; doi:10.7717/peerj.21530)
Supplement: Supplemental Information 3 — Partial sequence alignment of mCytB from F- and M-mtDNA, highlighting sequence divergence (grey shading) and the position of the primers used to amplify M-cytB and F-cytB in qPCR experiments. [file peerj-14-21530-s003.pdf]

qPCR primers for M-CytB

```
       118                                                                                                                 237
F-CytB gtaaacttaaacgcctggtgaaggtttggctctatactaggcttgtgtctggttatccaacttttgaggggtcttttattatcagcccactacactgctcatgaagacatggcatttgac
M-CytB attaacttaaacgcctggtggaggtttgggtctatactaggtttaaggttggtaatccaacttattagaggtcttttactgtcaattcactatactgcccacgaaagcatggcgtttgat
```

qPCR primers for F-CytB

```
       948                                                                                                                1067
F-CytB atacttaattcctacgcttcatacaggtaagtaccgaagtttatgcttctacccattgaatcaagtagtgttttgggtgttggttggaaggtttattagcctaacatgaattggtgctcg
M-CytB atacttaattcctagccttcatagaggaaagtatcgcagtttatgcttttacccatttaatcagttagtattctgggttttggtcgctagcctcatcagactaacgtgaattggtgcccg
```
