# Supplementary material for: Unstable mitochondrial heteroplasmy in Mytilus edulis primary cell cultures
Source: PeerJ. 2026 Jul 2;14:e21530. doi: 10.7717/peerj.21530 (PMC13333129; doi:10.7717/peerj.21530)
Supplement: Supplemental Information 4 — Raw derivative curves computed from the Melt Curve Data output of the qPCR instrument. Temperature range: 65-87 C. Blue lines: Mcytb target gene (wells A7-A12); red dashed: no-template control (NTC) for Mcytb (A13); green lines: 28SrRNA reference gene (D7-D12); grey dashed: NTC for 28SrRNA (D13). Panels outlined in orange indicate anomalous samples. M6: melting curve analysis revealed that the two primer pairs were loaded into transposed wells relative to all other individuals; wells could not be reassigned objectively due to an unexpected number of loaded wells. The sample was thus not considered in the analysis. F1: Mcytb wells showed broad non-specific amplification co-occurring with the NTC, leading to exclusion of this individual. [file peerj-14-21530-s004.pdf]

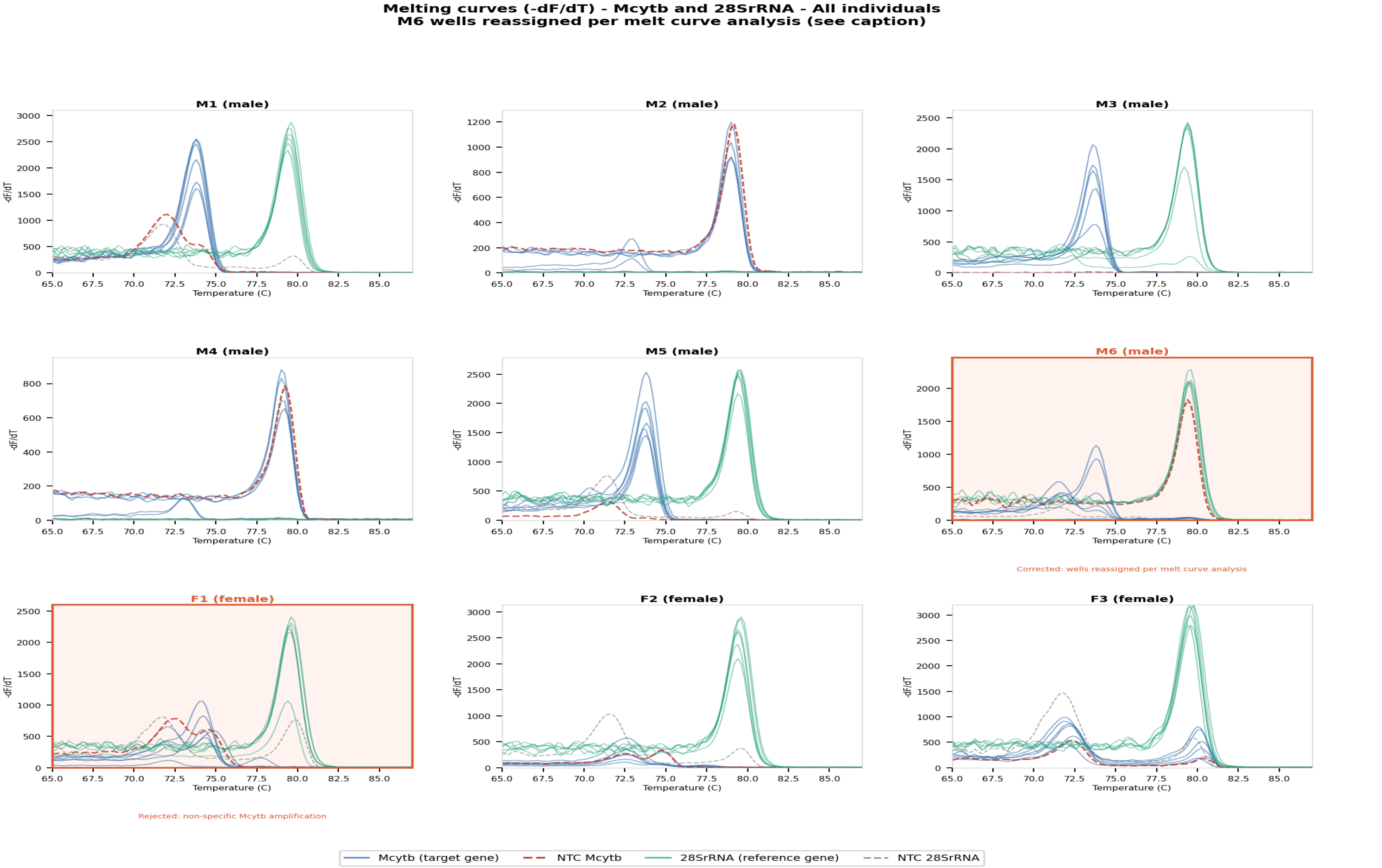

Supplementary Figure S2. Melting curves (-dF/dT) for Mcytb and 28SrRNA across all qPCR samples. Raw derivative curves computed from the Melt Curve Data output of the qPCR instrument. Temperature range: 65-87 C. Blue lines: Mcytb target gene (wells A7-A12); red dashed: no-template control (NTC) for Mcytb (A13); green lines: 28SrRNA reference gene (D7-D12); grey dashed: NTC for 28SrRNA (D13). Panels outlined in orange indicate anomalous samples. M6: melting curve analysis revealed that the two primer pairs were loaded into transposed wells relative to all other individuals; wells could not be reassigned objectively due to unexpected number of loaded wells. The sample was thus not considered in the analysis. F1: Mcytb wells showed broad non-specific amplification co-occurring with the NTC, leading to exclusion of this individual.
